# Sex-Specific Differences in the Revascularization of Grafted Pancreatic Islets

**DOI:** 10.3390/cells14171344

**Published:** 2025-08-29

**Authors:** Selina Wrublewsky, Annika Valerie Widmann, Caroline Bickelmann, Alex Rafacho, Leticia Prates Roma, Matthias W. Laschke, Emmanuel Ampofo

**Affiliations:** 1 Institute for Clinical & Experimental Surgery, Saarland University, PharmaScienceHub (PSH), 66421 Homburg, Germany; selina.wrublewsky@uks.eu (S.W.); matthias.laschke@uks.eu (M.W.L.); 2 Center for Gender Specific Biology and Medicine (CGBM), Saarland University, 66421 Homburg, Germany; 3 Laboratory of Investigation of Chronic Diseases, Biological Sciences Center, Department of Physiological Sciences, Florianópolis 88037-000, SC, Brazil; 4 Biophysics Department, Center for Human and Molecular Biology, Saarland University, 66421 Homburg, Germany

**Keywords:** islet transplantation, glucagon, type-1 diabetes, revascularization, sex, gender

## Abstract

Islet transplantation can improve glycemic control in a subset of patients with type 1 diabetes mellitus (T1DM). This therapeutic approach is often limited by scarcity of adequate donor islets and an insufficient revascularization capacity of grafted islets. Recent findings reveal that sex is an important determinant for the outcome of islet transplantation. However, it is still unknown how the biological sex of islet donors and recipients affects the revascularization of the grafts during the initial ischemic post-transplantation phase. In this study, we observed in a mouse dorsal skinfold chamber model a higher revascularization capacity of female islets transplanted in female or male recipient mice when compared to male islets transplanted in female or male recipients. To mimic the ischemic in vivo conditions ex vivo, we subjected isolated female and male islets to oxygen-glucose deprivation. Under these conditions female islets expressed and secreted significantly more glucagon (GCG). By a panel of functional angiogenesis assays, we could further demonstrate that GCG exhibits a strong pro-angiogenic function. This effect was pronounced in blood vessels as well as endothelial cells and pericytes of female origin due to a higher expression of GCG receptor. Taken together, these results not only confirm the clinical observation that transplantation of female islets improves the outcome of islet transplantation but also indicate that this is mediated by an accelerated GCG-driven islet engraftment.

## 1. Introduction

Islet transplantation is a clinical intervention for the treatment of a subset of patients with type 1 diabetes mellitus (T1DM). This procedure can substantially improve glycemic control and temporarily eliminate the need for exogenous insulin therapy [1]. However, an insufficient revascularization capacity of grafted islets and, thus, their massive loss during the initial post-transplantation phase represent major problems of this therapeutic approach [2,3,4,5,6,7].

It has been shown that the overall prevalence of diabetes is lower in women when compared to men [8]. Several experimental studies demonstrated that this is partially caused by the protective effect of estrogens on islet viability and endocrine function. For instance, the exposure of isolated islets to estrogens has been shown to protect them from diabetes-induced cellular stress conditions, including glucolipotoxicity, inflammation and oxidative stress [9,10,11]. Moreover, estrogens modulate insulin synthesis and insulin secretion [12], whereas glucagon (GCG) and somatostatin (SST) are not affected [13,14]. Furthermore, female sex hormones stimulate angiogenesis [15], which, in turn, promotes wound healing [16]. Accordingly, it can be assumed that the transplantation of islets in female recipients results in an improved outcome. In line with this view, Liu et al. [17] found in a preclinical study that mice treated with exogenous estrogen show an increased islet revascularization when compared to vehicle-treated controls. The transplantation of neonatal porcine islets also led to an improved glucose clearance compared to their male counterparts [18]. In addition, a retrospective clinical cohort study reported that transplanted islets survive longer in female recipients [19].

Beside the beneficial effect of female sex hormones on islet transplantation, it was reported that human recipients of islets from at least one female donor exhibit a prolonged graft survival when compared to recipients of islets from exclusively male donors [19]. In this context, Saber et al. [20] reported that stem cell-derived islets from female donors are superior to those from male donors after transplantation. This clearly indicates that not only female recipients but also islets of female origin favor the outcome of islet transplantation [21]. However, the underlying mechanisms remain elusive so far.

In the present study, we analyzed in a mouse model sex-dependent effects on the engraftment of islets during the initial post-transplantation phase. For this purpose, murine islets from female and male donors were transplanted on the striated muscle tissue within dorsal skinfold chambers of female and male mice. The revascularization of the grafts was analyzed by intravital fluorescence microscopy throughout an observation period of 14 days. In addition, we mimicked the ischemic in vivo conditions during the initial post-transplantation phase ex vivo by exposing female and male islets to oxygen-glucose deprivation and assessed their angiogenic and endocrine activity.

## 2. Material and Methods

### 2.1. Antibodies

The anti-CD31 antibody (DIA310) was purchased from Dianova (Hamburg, Germany). The anti-CD31-fluorescein isothiocyanate (FITC) (553372) antibody was purchased from BD Biosciences (San Jose, CA, USA). The anti-insulin antibody, the anti-beta-actin antibody (HRP-66009), anti-GCGR antibody (26784), anti-α-tubulin antibody (HRP-66031) and the anti-GCG (67286) antibody were purchased from Proteintech (Manchester, UK). The anti-somatostatin antibody (4770) was from Santa Cruz (Heidelberg, Germany). The anti-vascular endothelial growth factor (VEGF)-A (214424) and anti-VEGFR1 (32152) antibodies were from Abcam (Cambridge, UK). The anti-Akt1/2/3 antibody (11E7), anti-pAkt (4060) antibody, anti-ERK1/2 antibody (ab115799), anti-pERK1/2 antibody (ab50011), anti-mTOR antibody (2983) and anti-pmTOR antibody (5536) were from Cell Signaling (Frankfurt am Main, Germany). The anti-rabbit IgG Alexa Fluor 555 antibody (A-21429) and anti-rat IgG Alexa Fluor 488 antibody (A-21434) were purchased from Thermo Fisher Scientific (Karlsruhe, Germany). The peroxidase-labeled anti-rabbit antibody (NIF 824) and peroxidase-labeled anti-mouse antibody (NIF 825) were purchased from GE Healthcare (Freiburg, Germany).

### 2.2. Cell Culture

Primary human placenta-derived pericytes (hPC-PL) and human umbilical vein endothelial cells (HUVEC) (PromoCell, Heidelberg, Germany) were cultivated at 37 °C under a humidified 95%/5% (vol/vol) mixture of air and CO_2_. The cells were passaged at a split ratio of 1:3 after reaching confluence. All experiments were carried out with confluent cells between the third and seventh passage.

### 2.3. Oxygen-Glucose Deprivation

Isolated islets were cultivated in Dulbecco’s Modified Eagle Medium (DMEM) (1 g/L glucose) under hypoxic conditions (95% N_2_, 5% CO_2_ and 1% O_2_) for 16 h.

### 2.4. Western Blot Analysis

Whole-cell or tissue lysates were resolved on either a 7.5% or 12.5% SDS-PAGE gel and subsequently transferred to a polyvinylidene difluoride (PVDF) membrane. Membranes were blocked for 1 h in Tris-buffered saline (TBS) containing 0.1% Tween-20 and 5% dry milk, followed by overnight incubation at 4 °C with primary antibodies (1:500 dilution) in TBS (0.1% Tween-20) supplemented with 1% dry milk. After washing, membranes were incubated for 1 h with horseradish peroxidase (HRP)-conjugated secondary antibodies (anti-rabbit 1:500 or anti-mouse 1:2000). Protein detection was performed using luminol-based enhanced chemiluminescence (ECL; GE Healthcare). Uncropped Western blots are included as Supplementary Materials.

### 2.5. Tube Formation Assay

HUVEC or hPC-PL were seeded in a 96-well plate (1.5 × 10^4^ cells per well), which contained 50 µL Matrigel per well. The cells were exposed to 200 nM human GCG (G2044) from Sigma-Aldrich (Taufkirchen, Germany) or vehicle (ctrl). This concentration was already used in [22]. Phase-contrast light micrographs were taken after 7 h. Tube formation was assessed by counting the number of tube meshes—defined as regions fully enclosed by endothelial tubes—per high-power field (HPF), using ImageJ software (version 1.54m) (U.S. NIH).

### 2.6. Cell Growth

HUVEC or hPC-PL (2.5 × 10^4^ cells per well) were seeded in a 24-well plate and cultivated overnight. Then, the cells were exposed to GCG (200 nM) or vehicle (ctrl) for 24 h, 48 h or 72 h. Thereafter, the cells were detached, centrifuged and suspended with fresh culture medium. Aliquots of the suspended cells were stained with trypan blue (0.4%) and counted.

### 2.7. Flow Cytometry

Islets were dispersed into single cells by Accutase. Subsequently, the cells were washed in phosphate-buffered saline (PBS) and incubated with phycoerythrin (PE)-labeled primary antibodies and PE-labeled IgG control antibodies for 1 h at room temperature. Then, the cells were washed in PBS and the percentage of CD31-positive cells of 5000 cells was analyzed by flow cytometry using a FACSLyrics (BD Biosciences).

### 2.8. Quantitative Real Time-Polymerase Chain Reaction (qRT-PCR)

Total RNA was extracted from pancreatic islets using the QIAzol lysis reagent (Qiagen, Hilden, Germany). Complementary DNA (cDNA) was synthesized from 1 μg of total RNA utilizing the QuantiNova Reverse Transcription Kit (Qiagen, Hilden, Germany), following the manufacturer’s protocol. Quantitative real-time PCR (qRT-PCR) was performed using ORA qPCR Green ROX L Mix (highQu, Kraichtal, Germany). Data were acquired and analyzed with the MiniOpticon Real-Time PCR System (Bio-Rad, Feldkirchen, Germany). Murine β-actin was used as the internal reference gene for mRNA normalization. Forward and reverse primers were applied at a final concentration of 700 nM, dissolved in RNase/DNase-free H_2_O. Primer sequences for qPCR were coded as follows: mouse Ins-1 forward 5′-AACAACTGGAGCTGGGAGGAAG-3′, reverse 5′-GGTGCAGCACTGATCCACAATG-3′; mouse Ins-2 forward 5′-GCAGCACCTTTGTGGTTCC-3′, reverse 5′-CTTGTGGGTCCTCCACTTC-3′; mouse Vegf-A forward 5′-GCTGTACCTCCACCATGCCAAG-3′, reverse 5′-CGCACTCCAGGGCTTCATCG’; mouse CD31 forward 5′-CACAGAAGTGGAAGTGTCCT-3′, reverse 5′-ACCTTCCGGATCTCACTGT-3′; mouse SST forward 5′-CCCAACCAGACAGAGAATGA-3′, reverse 5′-ACAGGATGTGAATGTCTTCCA-3′; mouse GCG forward 5′-TGGACTCCCGCCGTGCTCAAG-3′, reverse 5′-CCTTTGCTGCCTGGCCCTCC-3′; mouse GCGR forward 5′-CAATGCCACCACAACCTAAGCC-3′, reverse 5′-GGCAGGAAATGTTGGCAGTGGT-3′; mouse β-actin forward 5′-CCTAGGCACCAGGGTGTGAT-3′, reverse 5′-TCTCCATGTCGTCCCAGTTG-3′.

### 2.9. Enzyme-Linked Immunosorbent Assay (ELISA)

The amount of secreted insulin was measured by a mouse insulin ELISA kit. For this purpose, 10 isolated islets were washed with Krebs Ringer Buffer (KRB) (115 mM NaCl, 4.7 mM KCl, 1.28 mM CaCl_2_, 1.2 mM MgSO_4_, 0.1% BSA) and incubated for 1 h at 37 °C and 5% CO_2_. The supernatants were discarded, and the islets were incubated for 30 min in KRB containing 16.5 mM glucose. The supernatants were collected and the amount of secreted insulin was determined by using an insulin ELISA kit according to the manufacturer’s protocol.

The amount of secreted GCG was measured by a GCG ELISA kit. For this purpose, 20 islets were cultivated in KRB (115 mM NaCl, 4.7 mM KCl, 1.28 mM CaCl_2_, 1.2 mM MgSO_4_, 0.1% BSA, 25 mM glucose) for 1 h at 37 °C and 5% CO_2_. Subsequently, the buffer was removed and the islets were cultivated in KRB containing 0.5 mM glucose for 2 h. The supernatants were collected and the amount of secreted GCG was determined by using a GCG ELISA kit according to the manufacturer’s protocol.

The amount of secreted SST was measured by a SST ELISA kit. For this purpose, 20 islets were cultivated in KRB (140 mM NaCl, 3.6 mM KCl, 2.6 mM CaCl_2_H_2_O, 0.5 mM MgSO_4_7H_2_O, 0.5 mM NaH_2_PO_4_, 2 mM NaHCO_3_, 5 mM HEPES, 1 mM glucose) for 1 h at 37 °C and 5% CO_2_. Subsequently, the buffer was removed, and the islets were cultivated in KRB (70 mM NaCl, 70 mM KCl, 2.6 mM CaCl_2_H_2_O, 0.5 mM MgSO_4_7H_2_O, 0.5 mM NaH_2_PO_4_, 2 mM NaHCO_3_, 5 mM HEPES, 20 mM glucose) for 2 h. The supernatants were collected, and the amount of secreted SST was determined by using a SST ELISA kit according to the manufacturer’s protocol.

### 2.10. Animals

C57BL/6J mice were purchased from the Jackson Laboratory. Female and male C57BL/6J mice with a body weight of 25–30 g served as donors for islet isolation. Female and male C57BL/6J mice with a body weight of 23–25 g were used for the dorsal skinfold chamber model. Animals were maintained on a standard 12/12 h day/night cycle. Water and standard pellet chow (Altromin, Lage, Germany) were provided ad libitum.

The study was approved by the Institutional Review Board (or Ethics Committee) of State Office for Consumer Protection, Saarbrücken, Germany; permission numbers: 45/2018, 30/2019 and 08/2024.

### 2.11. Isolation of Pancreatic Islets

Mice were anesthetized via intraperitoneal (i.p.) injection of ketamine (100 mg/kg body weight) and xylazine (12 mg/kg body weight). After cervical dislocation and a midline laparotomy, the pancreatic duct was perfused with 1 mg/mL collagenase NB 8 containing 25 µL/mL neutral red solution. Pancreatic islets were then isolated as previously described in detail [23].

### 2.12. Preparation of the Dorsal Skinfold Chamber and Islet Transplantation

Mice were anesthetized with an intraperitoneal (i.p.) injection of ketamine (100 mg/kg body weight) and xylazine (12 mg/kg body weight), and the dorsal skinfold chamber was implanted. In brief, two symmetrical titanium frames were prepared on the extended dorsal skinfold of the anesthetized mice, creating a double layer of skin. One layer—comprising the skin, subcutaneous tissue, and retractor muscle—was surgically removed within a circular area of 15 mm in diameter. This exposed area was then covered with a removable glass coverslip secured by a snap ring, enabling direct microscopic observation of the chamber’s microvasculature. Following implantation, the animals were allowed a 48 h recovery period.

After recovery, the mice were re-anesthetized using the same ketamine/xylazine regimen. The coverslip was removed, and the tissue surface was rinsed with saline. Subsequently, eight isolated islets were transplanted onto the exposed striated muscle tissue. The chamber was then resealed with a new coverslip to facilitate repeated intravital fluorescence microscopy.

### 2.13. Intravital Fluorescence Microscopy

Anesthetized dorsal skinfold chamber-equipped mice received a retrobulbary intravenous injection of 0.05 mL FITC-labeled dextran (5%) for plasma staining [24] on day 0 as well as day 3, 6, 10 and 14 after islet transplantation. The dorsal skinfold chamber was positioned under a fluorescence microscope (Zeiss, Oberkochen, Germany) and the microscopic images were recorded by CapImage system (version 10; Zeintl, Heidelberg, Germany). The revascularized area (mm^2^) and the functional microvessel density (cm/cm^2^) of islets were assessed. In addition, we measured the diameter (µm) and centerline red blood cell (RBC) velocity (µm/s) of 4–8 individual microvessels within the grafts [24]. Moreover, the take rate (%), i.e., the number of engrafted islets containing blood-perfused microvessels on day 14 in relation to the number of transplanted islets on day 0, was determined.

### 2.14. Isolation of Microvascular Fragments (MVF) and Spheroid Sprouting Assay

Anesthetized mice were euthanized by cervical dislocation. Subsequently, MVF were mechanically and enzymatically isolated (collagenase IAS) from isolated visceral fat pads, as described previously in detail [25]. MVF were cultivated in DMEM (10% (*v*/*v*) fetal calf serum (FCS), 100 U/mL penicillin and 0.1 mg/mL streptomycin) at 37 °C and 5% CO_2_. MVF spheroids were formed by the liquid overlay technique in a 96-well plate. For this purpose, 750 MVF were seeded per well and cultivated for 5 days to allow the formation of a spheroid at 37 °C under a humidified 95% to 5% (*v*/*v*) mixture of air and CO_2_. After 5 days, the spheroids were harvested. The angiogenic activity of the MVF spheroids was assessed by a sprouting assay. For this purpose, MVF spheroids were resuspended in a collagen solution and transferred into 24-well plates [26]. After 45 min, the collagen gel was covered with DMEM (10% (*v*/*v*) FCS, 100 U/mL penicillin and 0.1 mg/mL streptomycin) containing GCG (200 nM) or vehicle (ctrl) and the spheroids were incubated for 3 days at 37 °C and 5% CO_2_ for daily analyses. The sprouting was visualized by a BX60F microscope (Olympus, Hamburg, Germany) and assessed by measuring the sprouting area with the FIJI software (version 1.54m) (NIH).

### 2.15. Immunohistochemistry

The dorsal skinfold chamber tissue was fixed overnight in 4% paraformaldehyde (PFA). The PFA-fixed samples were embedded in paraffin and 3-μm-thick sections were cut. The sections were stained with antibodies against insulin, GCG, and SST and visualized by their corresponding secondary antibodies. Cell nuclei were stained with Hoechst 33342. The sections were analyzed by means of a BX60F microscope (Olympus). The quantification of positively stained cells was done by the FIJI software (NIH) and is given in % of all islet cells.

### 2.16. Statistical Analysis

All in vitro and ex vivo experiments were reproduced at least three times. For the in vivo study, we used 8 animals per group. After testing the data for normal distribution and equal variance, differences between two groups were assessed by the unpaired Student’s *t*-test. To test differences between multiple groups, two-way ANOVA was applied by means of the GraphPad Prism software (version 10). All values are expressed as mean ± SD (standard deviation). Statistical significance was accepted for *p* < 0.05.

## 3. Results

### 3.1. The Revascularization of Female Islets Is Accelerated When Compared to Male Islets

**Figure 1:**
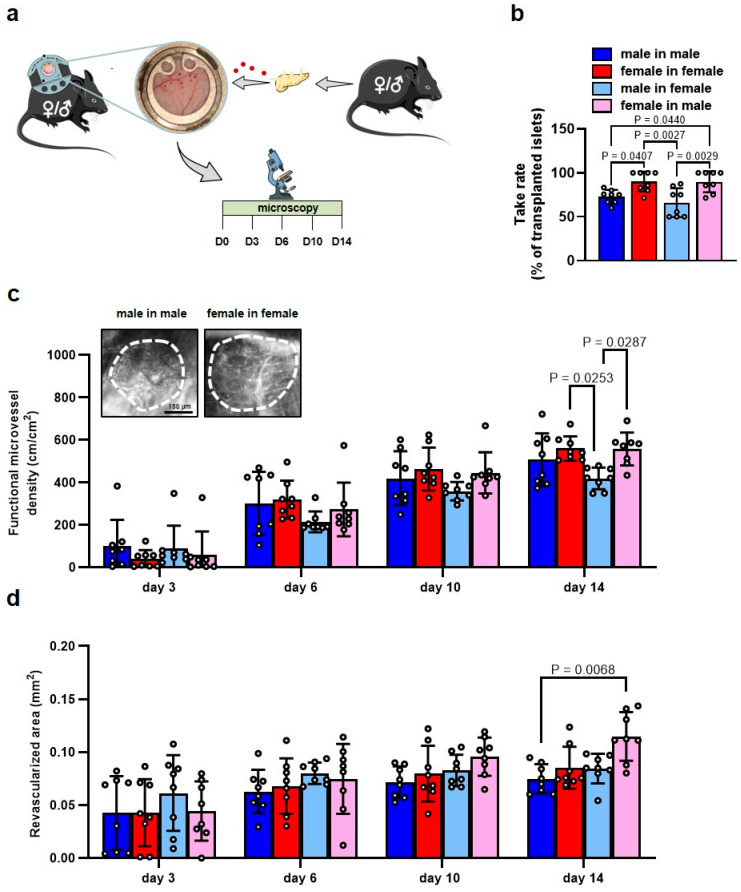
Revascularization of transplanted islets. (**a**) Schematic illustration of the experimental setting. Islets from female or male donor mice were transplanted on the striated muscle tissue within the dorsal skinfold chambers of female or male recipients. Intravital fluorescence microscopy was performed on days 0, 3, 6, 10 and 14 after islet transplantation. (**b**) Take rate of islets (% of transplanted islets; *n* = 8 each) on day 14 after islet transplantation. Mean ± SD. (**c**) Functional microvessel density (cm/cm^2^) of islets of the different groups (*n* = 8 each). Mean ± SD. Upper panel: Representative intravital fluorescent microscopic images of transplanted male islets in male recipients and female islets in female recipients on day 14. FITC-labeled dextran was used for the visualization of blood-perfused microvessels. The border of the grafts is marked by broken lines. Scale bar: 150 μm. (**d**) Revascularized area (mm^2^) of islets of the different groups (*n* = 8 each). Mean ± SD.

To study whether the well-known improved outcome of clinically transplanted female islets is caused by an ameliorated revascularization, we transplanted isolated islets from female and male donor mice onto the striated muscle tissue within dorsal skinfold chambers of female and male recipient mice. Thereafter, we analyzed the development of new blood vessels within the grafts by means of repeated intravital fluorescence microscopy (Figure 1a). Of interest, we detected a markedly higher take rate of islets of female origin in female and male recipients when compared to transplanted islets of male origin in male and female recipients (Figure 1b). In line with these results, the application of FITC-labeled dextran revealed a higher functional microvessel density and a larger revascularized area of the grafts on day 14 in mice receiving female islets (Figure 1c,d). The additional measurement of microhemodynamic parameters showed no sex-dependent differences in the diameter of individual intra-islet blood vessels (Supplementary Figure S1a). However, we detected a significantly higher centerline RBC velocity in microvessels of transplanted female islets on day 14 (Supplementary Figure S1b).

**Figure 2:**
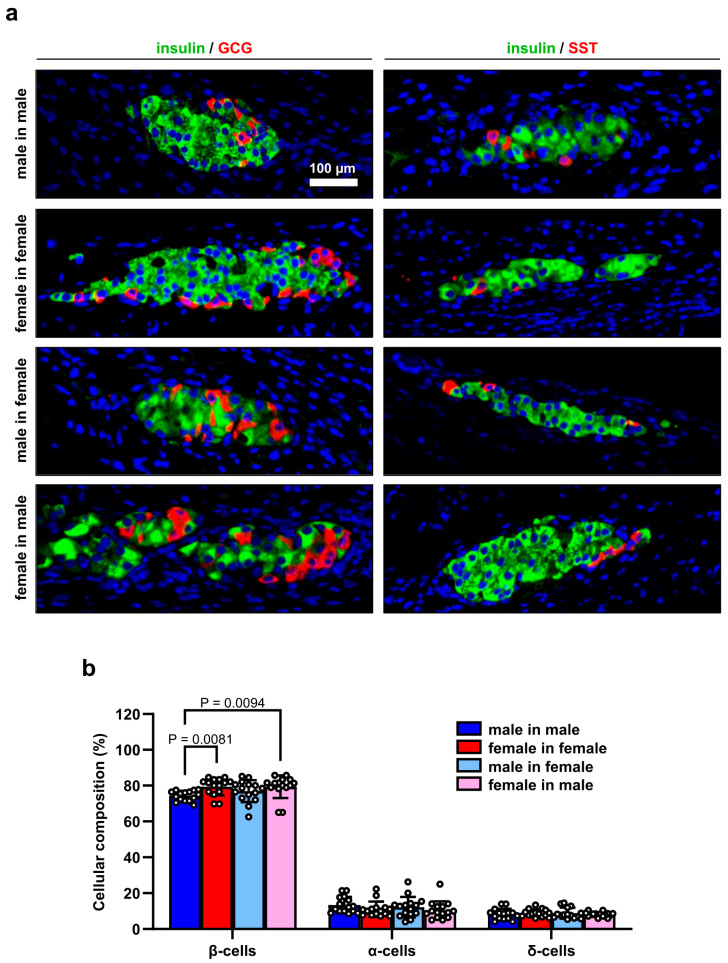
Cellular composition of transplanted islets. (**a**) Representative immunofluorescent stainings of insulin/GCG and insulin/SST in transplanted islets of the different groups on day 14. Cell nuclei were stained with Hoechst 33,342 (blue). Scale bar: 100 µm. (**b**) Insulin- (β-cells), GCG- (α-cells) and SST- (δ-cells) positive cells in transplanted islets of the different groups on day 14. Data are given in % of all islet cells (*n* = 15 islets each). Mean ± SD.

To characterize the endocrine cellular composition of the islets on day 14 after transplantation, we excised the grafts and examined the expression of insulin, GCG and SST by immunohistochemistry. Of interest, we detected a slightly, but significantly higher fraction of insulin-positive β-cells in the groups of female transplants in female as well as male recipients when compared to the group of male transplants in male recipients (Figure 2a,b). In contrast, we did not observe any differences in the fractions of α- and δ-cells between the groups (Figure 2a,b). Taken together, these results show that the revascularization capacity of female islets is superior to that of male islets after transplantation, which, in turn, may improve β-cell survival.

### 3.2. Ischemic Female Islets Express More VEGF-A and GCG When Compared to Male Islets

**Figure 3:**
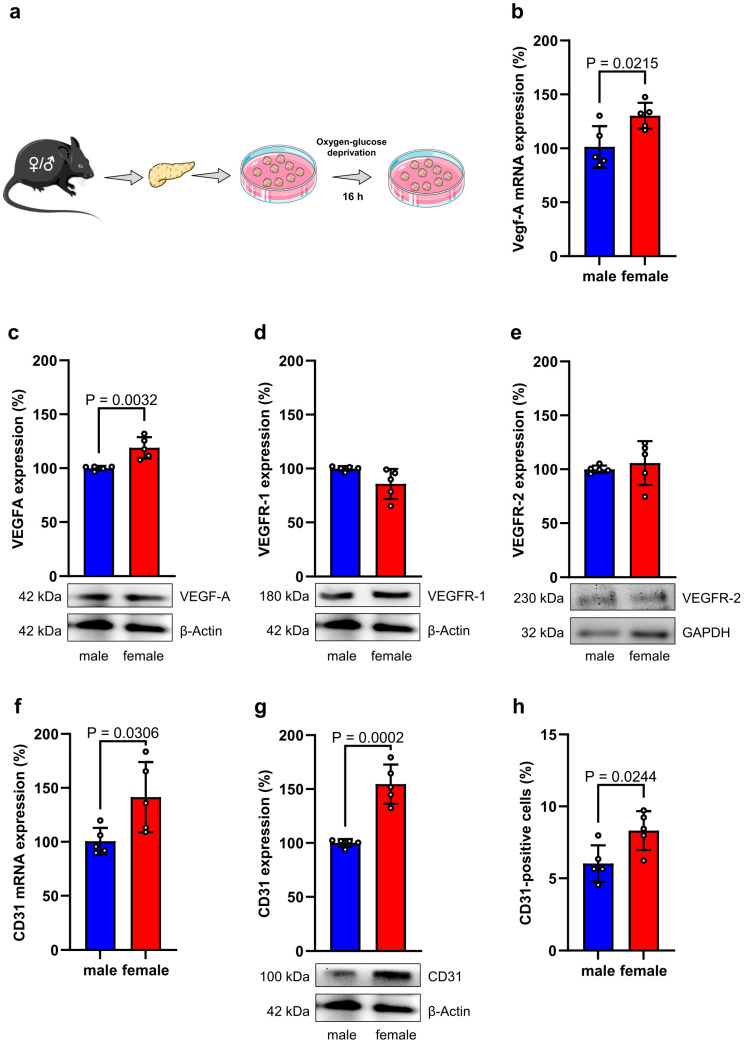
Angiogenic capacity of isolated islets. (**a**) Schematic illustration of the experimental setting. Islets were isolated from female and male mice and subjected to oxygen-glucose deprivation for 16 h. Subsequently, the islets were collected and prepared for protein expression and secretion analyses. (**b**) VEGF-A mRNA expression in isolated female and male islets subjected to oxygen-glucose deprivation for 16 h. Data are expressed in % of male islets (*n* = 5 each). Mean ± SD. (**c**–**e**) VEGF-A (**c**), VEGFR-1 (**d**) and VEGFR-2 (**e**) protein expression in isolated female and male islets subjected to oxygen-glucose deprivation for 16 h. Data are expressed in % of male islets (*n* = 5 each). Mean ± SD. Lower panel: Representative Western blots of VEGF-A (**c**), VEGFR-1 (**d**) and VEGFR-2 (**e**), β-actin and GAPDH from whole cell extracts of these islets. (**f**) CD31 mRNA expression in isolated female and male islets subjected to oxygen-glucose deprivation for 16 h. Data are expressed in % of male islets (*n* = 5 each). Mean ± SD. (**g**) CD31 protein expression in isolated female and male islets subjected to oxygen-glucose deprivation for 16 h. Data are expressed in % of male islets (*n* = 5 each). Mean ± SD. Lower panel: Representative Western blots of CD31 and β-actin from whole cell extracts of these islets. (**h**) CD31 surface protein expression in isolated female and male islets subjected to oxygen-glucose deprivation for 16 h, as assessed by flow cytometry (*n* = 5 each). Mean ± SD.

VEGF-A is expressed in β-cells and plays a crucial role in the development of islet revascularization during the initial ischemic post-transplantation phase [27]. To mimic these conditions ex vivo, we subjected isolated female and male islets to oxygen-glucose deprivation for 16 h (Figure 3a). By this, we found a higher gene and protein expression of VEGF-A in female islets (Figure 3b,c). In contrast, the corresponding receptors of VEGF-A, VEGFR-1 and VEGFR-2, were not differently expressed in female and male islets (Figure 3d,e). We additionally analyzed the expression of the vascular surface protein CD31. Of note, we detected a significantly higher CD31 gene and protein expression in isolated female islets exposed to hypoxia and low glucose (Figure 3f–h).

**Figure 4:**
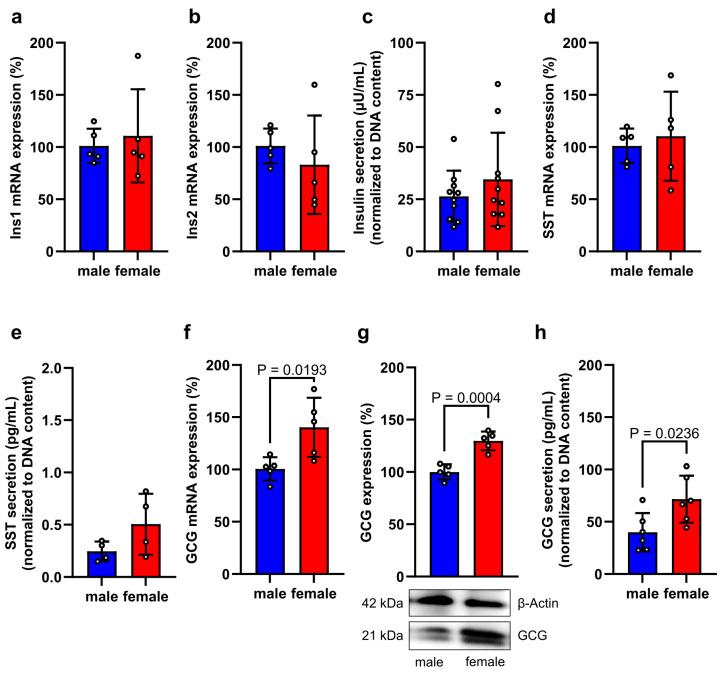
Endocrine activity of isolated islets. (**a**,**b**) Ins1 (**a**) and Ins2 (**b**) mRNA expression in isolated female and male islets subjected to oxygen-glucose deprivation for 16 h. Data are expressed in % of male islets (*n* = 5 each). Mean ± SD. (**c**) Insulin secretion (µU/mL) from isolated female and male islets subjected to oxygen-glucose deprivation for 16 h (*n* = 10 each). Mean ± SD. (**d**) SST mRNA expression in isolated female and male islets subjected to oxygen-glucose deprivation for 16 h. Data are expressed in % of male islets (*n* = 5 each). Mean ± SD. (**e**) SST secretion (pg/mL) from isolated female and male islets subjected to oxygen-glucose deprivation for 16 h (*n* = 4 each). Mean ± SD. (**f**) GCG mRNA expression in isolated female and male islets subjected to oxygen-glucose deprivation for 16 h. Data are expressed in % of male islets (*n* = 5 each). Mean ± SD. (**g**) GCG protein expression in isolated female and male islets subjected to oxygen-glucose deprivation for 16 h. Data are expressed in % of male islets (*n* = 5 each). Mean ± SD. Lower panel: Representative Western blots of GCG and β-actin from whole cell extracts of these islets. (**h**) GCG secretion (pg/mL) from isolated female and male islets subjected to oxygen-glucose deprivation for 16 h (*n* = 6 each). Mean ± SD.

We additionally studied the sex-dependent expression and secretion of endocrine hormones in isolated female and male islets. Previous studies already reported that under physiological conditions female islets secrete more insulin when compared to male islets [28,29,30]. However, during oxygen-glucose deprivation, we did not detect any significant differences in the expression and secretion of insulin as well as SST between female and male islets (Figure 4a–e). In contrast, we detected a markedly altered sex-dependent GCG expression. In fact, we found that isolated female islets exposed to hypoxia and low glucose exhibit a significantly higher GCG gene and protein expression (Figure 4f,g). Accordingly, these islets also secreted more GCG when compared to male islets (Figure 4h).

### 3.3. GCG Promotes Angiogenesis of Blood Vessels of Female Origin

**Figure 5:**
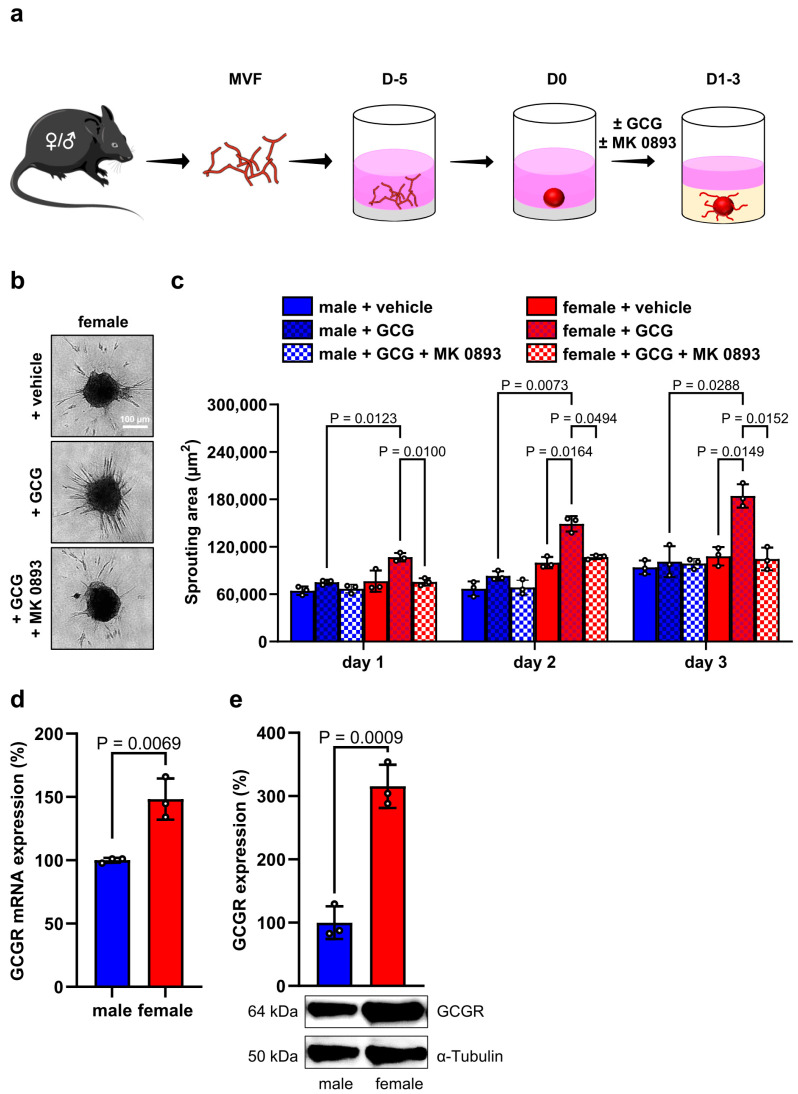
Effect of GCG on the angiogenic activity of MVF. (**a**) Schematic illustration of the experimental setting. MVF from female and male mice were fused to spheroids by means of the liquid overlay technique and cultured for 5 days. On day 0, the spheroids were embedded into a collagen matrix and exposed to GCG alone or GCG and MK 0893. The sprouting activity was assessed on days 1, 2 and 3. (**b**) Representative images of female MVF spheroids exposed to GCG, GCG and MK 0893 or vehicle as control. Scale bar: 100 µm. (**c**) Sprouting area (µm^2^) of female and male MVF spheroids exposed to GCG, GCG and MK 0893 or vehicle at the indicated days (*n* = 3 each). Mean ± SD. (**d**) GCGR mRNA expression in female and male MVF spheroids. Data are expressed in % of male MVF spheroids (*n* = 3 each). Mean ± SD. (**e**) GCGR protein expression in female and male MVF spheroids. Data are expressed in % of male MVF spheroids (*n* = 3 each). Mean ± SD. Lower panel: Representative Western blots of GCGR and α-tubulin from whole cell extracts of these MVF spheroids.

We hypothesized that the herein observed increased GCG secretion from female islets stimulates their graft revascularization. To test this, we performed an ex vivo spheroid sprouting assay. For this purpose, MVF were isolated from female or male mice and fused to compact spheroids, which were subsequently exposed to recombinant GCG alone or in combination with the specific GCG receptor (GCG-R) inhibitor MK 0893 (Figure 5a). We found that GCG significantly increases the sprouting activity of female MVF spheroids over the period of 3 days (Figure 5b,c). Of note, this pro-angiogenic effect was abolished by the simultaneous treatment of the spheroids with MK 0893 (Figure 5b,c). In contrast, GCG did not affect the sprouting capacity of male MVF spheroids (Figure 5c). Hence, we assumed that GCG signaling transduction is more pronounced in blood vessels of female origin. In line with this assumption, we detected a higher GCGR gene and protein expression in female MVF spheroids (Figure 5d,e).

**Figure 6:**
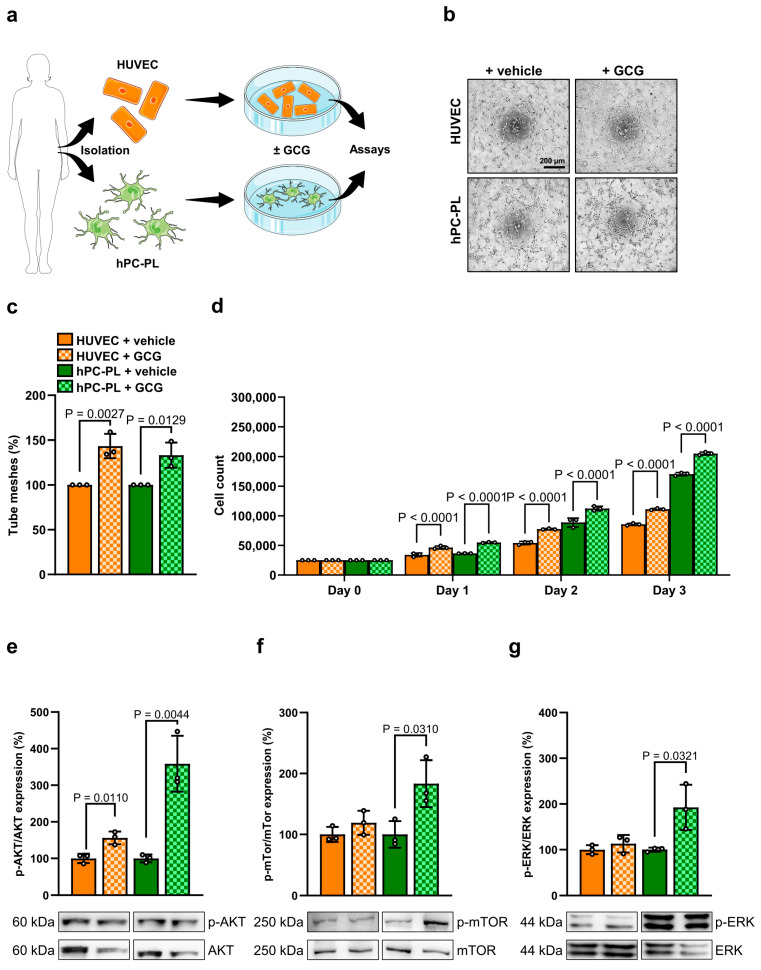
Effect of GCG on the angiogenic activity of female endothelial cells and pericytes. (**a**) Schematic illustration of the experimental setting. Endothelial cells (HUVEC) and pericytes (hPC-PL) were isolated from human female donors, exposed to GCG or vehicle as control and their angiogenic activity was assessed by different assays. (**b**) Tube formation assays were performed with HUVEC and hPC-PL exposed to GCG or vehicle. The formation of tube-like structures was analyzed 7 h after seeding (representative images). Scale bar: 200 µm. (**c**) Quantitative analysis of the number of tube meshes (per HPF) after 7 h. Data are expressed in % of vehicle (*n* = 3 each). Mean ± SD. (**d**) HUVEC and hPC-PL were exposed to GCG or vehicle and the cell number was determined on days 0, 1, 2 and 3. Data are expressed in % of vehicle on day 0 (*n* = 3 each). Mean ± SD. (**e**–**g**) p-Akt/Akt (**e**), p-mTOR/mTOR-1 (**f**) and p-ERK/ERK-2 (**g**) protein expression in HUVEC and hPC-PL exposed to GCG or vehicle. Data are expressed in % vehicle (*n* = 3 each). Mean ± SD. Lower panel: Representative Western blots of p-Akt/Akt (**e**), p-mTOR/mTOR-1 (**f**) and P-ERK/ERK-2 (**g**) from whole cell extracts of HUVEC and hPC-PL exposed to GCG or vehicle.

To further elucidate the pro-angiogenic effect of GCG on angiogenesis, we exposed endothelial cells and pericytes from human female donors to GCG and studied their potential to form tube-like structures as well as to proliferate (Figure 6a). HUVEC and hPC-PL are capable of forming tube-like structures (Figure 6b). Of interest, this process was more pronounced when the cells are exposed to GCG (Figure 6b,c). The additional analysis of cell proliferation demonstrated a minor, but significantly increased proliferation of both vascular cell types treated with GCG when compared to vehicle-treated controls (Figure 6d). Finally, we assessed pAkt, pmTOR and pERK expression as indicator for the underlying signaling pathways activated by GCG. This analysis showed that GCG activates Akt signaling in HUVEC and hPC-PL (Figure 6e), whereas a significantly elevated mTOR and ERK signaling was solely found in hPC-PL (Figure 6f,g).

## 4. Discussion

It is well known that sex has a crucial impact on the clinical outcome of islet transplantation [21]. In this context, Alejandro and colleagues reported that T1DM patients transplanted with islets from at least one female donor exhibit prolonged graft survival when compared to recipients of exclusively male islet donors [19]. In addition, they demonstrated that female recipients also show prolonged survival when compared to male recipients following islet transplantation of at least one female donor [19]. However, so far, the existing preclinical T1DM rodent models are not suitable to analyze the underlying mechanisms of these improved outcomes. For instance, the incidence of spontaneous diabetes in nonobese diabetic (NOD) mice is ~70% for females and ~30% for males [31]. This may be caused by the dysregulated immune system resulting in insulitis characterized by leukocytic infiltration of the pancreatic islets. However, the exact molecular process is still elusive [32,33]. Moreover, female C57BL/6 mice and other mouse strains are partially resistant to streptozotocin (STZ)-induced diabetes [10] and, thus, preclinical research related to diabetic complications commonly use males [34,35].

In the present study, we used a dorsal skinfold chamber model in non-diabetic mice. This model represents an attractive approach for monitoring the revascularization of various tissue transplants, including pancreatic islets, over time [25,34,36,37,38]. By means of this model we analyzed the revascularization of male islets transplanted into male or female recipients as well as female islets transplanted into female or male recipients. Our in vivo results clearly demonstrated a higher take rate and revascularized area of transplanted islets of female origin in female as well as male recipients. This is in line with abovementioned clinical observations [19,21] and indicates the superior function of female islets in the outcome of islet transplantation.

To obtain further insights into sex-dependent mechanisms underlying the revascularization of transplanted islets, we studied the expression and release of endocrine hormones in female and male islets. It is controversially discussed whether insulin expression and secretion depend on sex. Some studies reported that isolated islets from females consist of a higher fraction of β-cells and secrete more insulin when compared to male islets [28,29,30]. Others demonstrated that there is no effect of female sex hormones on the endocrine function of islets [39,40,41]. In the present study, we did not detect any differences in the expression and secretion of insulin during oxygen-glucose deprivation. SST, which has been reported to affect angiogenesis depending on its concentration [42], was also not affected. However, we found a significantly higher expression of GCG in isolated female islets when compared to male islets. This is in contrast to the results of Handgraaf et al. [13], showing that estradiol directly acts on isolated α cells through all estrogen receptors. This leads to a changed maturation of pro-GCG-derived peptides to increase GLP-1 and decreased GCG gene expression [13]. In the present study, islets are subjected to low glucose and hypoxia. The latter has been reported to enhance plasma GCG levels especially in females by increased metabolic stress [43,44]. Based on our results and these findings, it is conceivable that hypoxic stress is superior to female sex hormones and, thus, increases GCG gene expression.

Based on these findings, we speculated that GCG may stimulate the revascularization of transplanted female islets by inducing angiogenesis at the recipient site. Wang et al. [45] reported that GCG promotes angiogenesis under hypoxic conditions. However, the underlying mechanism is still not known. It can be assumed that this is due to a direct binding of GCG to GCG-R on vascular cells. In addition, it is conceivable that the pro-angiogenic effect of GCG is mediated via its binding to the glucagon-like peptide-1 receptor (GLP-1R), because GCG may act as a nonconventional GLP-1R agonist [46,47,48]. We found that GCG markedly activates angiogenic pathways in female endothelial cells and pericytes, resulting in enhanced blood vessel sprouting. Moreover, we detected a strong sprouting out of GCG-stimulated female MVF spheroids. However, this was not the case for male MVF spheroids. This may be explained by a sex-dependent GCG-mediated signaling transduction. In line with this view, we measured an elevated gene expression of GCG-R in female MVF spheroids indicating that the herein observed in vitro and ex vivo effects are indeed triggered by GCG.

Our in vivo results showed not only an improved revascularization of transplanted female islets in female recipients but also in male recipients. This contrasts with our in vitro and ex vivo results demonstrating a GCG-R-mediated signaling solely in female MVF. This discrepancy may be explained by the herein observed sex-dependent VEGF-A expression. VEGF-A is a predominant regulator of islet angiogenesis and revascularization. It has already been reported that the overexpression of VEGF-A or the exposure of islets to VEGF-A significantly increases graft revascularization in the early post-transplantation period in both mouse and human systems [49,50]. In the present study, we detected a significantly higher expression of VEGF-A in female islets. Hence, it is conceivable that this upregulated VEGF-A expression ameliorates their revascularization in male recipients by stimulating angiogenesis at the recipient site.

A limitation of our study is that we used non-diabetic animals. Hence, it remains unclear whether the beneficial effect of GCG on graft revascularization is also present in diabetic animals. Moreover, we transplanted islets onto the striated muscle tissue of mice, which allows the visualization of the engraftment. However, this is not the standard site for islet transplantation. Under clinical conditions islets are transplanted via the portal vein system into the liver.

## 5. Conclusions

In the present study, we found that female islets exhibit an accelerated revascularization after transplantation in female as well as in male recipient mice. This finding is in line with clinical data showing that recipients of islets from female donors exhibit a prolonged graft survival when compared to recipients of islets from exclusively male donors [19]. Hence, islets from female donors should be preferred to improve the outcome of islet transplantation. However, in clinical practice this is not possible due to several reasons, including the scarcity of appropriate organ donors and human leukocyte antigen (HLA)-matching [51]. Moreover, islet transplant recipients typically require islet infusions from up to 4 donor pancreases to achieve the goal of receiving ≥10,000 islet equivalent units (IEQ)/kg [52,53].

## Data Availability

The data supporting this study are included in the article or Supplementary Materials.

## Supplementary Materials

The following supporting information can be downloaded at: https://www.mdpi.com/article/10.3390/cells14171344/s1, Figure S1: Uncropped Western blots of VEGF-A and β-actin from whole cell extracts of islets; Figure S2: Uncropped Western blots of VEGFR-1 and β-actin from whole cell extracts of islets; Figure S3: Uncropped Western blots of VEGFR-2 and GAPDH from whole cell extracts of islets; Figure S4: Uncropped Western blots of CD31 and β-actin from whole cell extracts of islets; Figure S5: Uncropped Western blots of GCG and β-actin from whole cell extracts of islets; Figure S6: Uncropped Western blots of GCGR and α-tubulin from whole cell extracts of islets; Figure S7: Uncropped Western blots of p-AKT and AKT from whole cell extracts of HUVEC; Figure S8: Uncropped Western blots of p-AKT and AKT from whole cell extracts of hPC-PL; Figure S9: Uncropped Western blots of p-mTOR and mTOR from whole cell extracts of HUVEC; Figure S10: Uncropped Western blots of p-mTOR and mTOR from whole cell extracts of hPC-PL; Figure S11: Uncropped Western blots of p-ERK and ERK from whole cell extracts of HUVEC; Figure S12: Uncropped Western blots of p-ERK and ERK from whole cell extracts of hPC-PL.
