# Supplementary material for: Sex-Specific Differences in the Revascularization of Grafted Pancreatic Islets
Source: Cells. 2025 Aug 29;14(17):1344. doi: 10.3390/cells14171344 (PMC12428581; doi:10.3390/cells14171344)
Supplement: Supplementary file 1 [file cells-14-01344-s001.zip › cells-3715472-supplementary.pdf]

Supplementary Figure S1

VEGFA

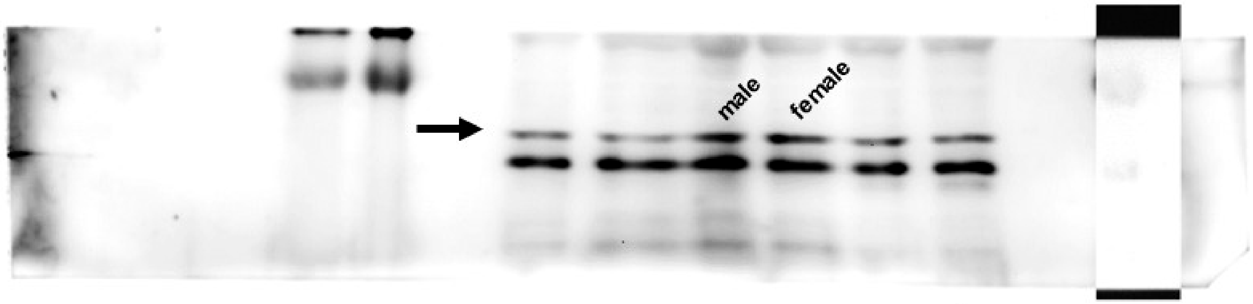

(a)

ß-actin

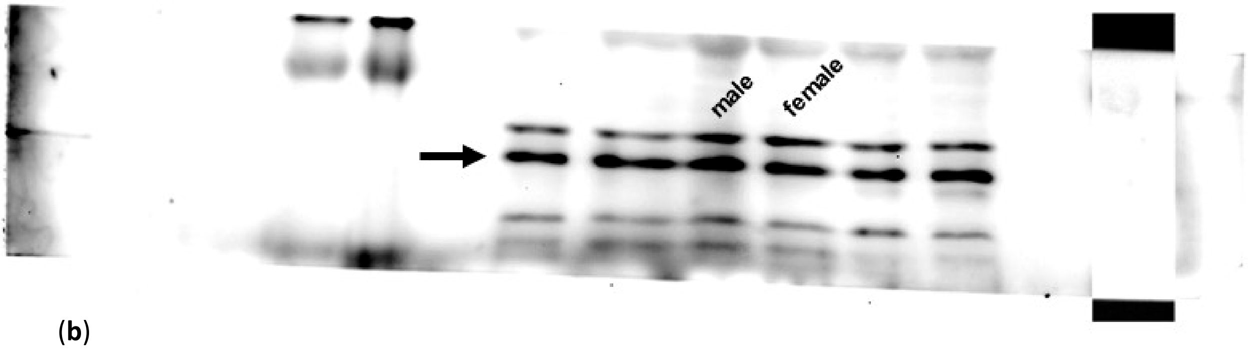

(b)

Supplementary Figure S1: (**a**,**b**) Uncropped Western blots of VEGF-A and β-actin from whole cell extracts of islets.

Supplementary Figure S2

VEGFR-1

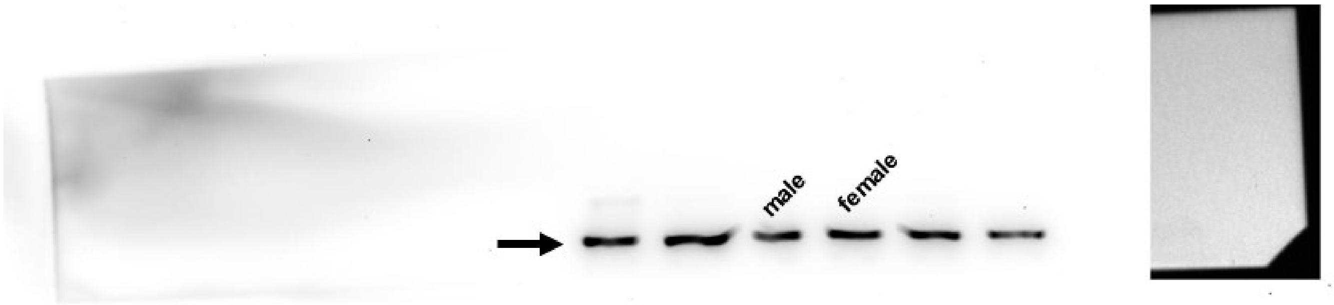

ß-actin

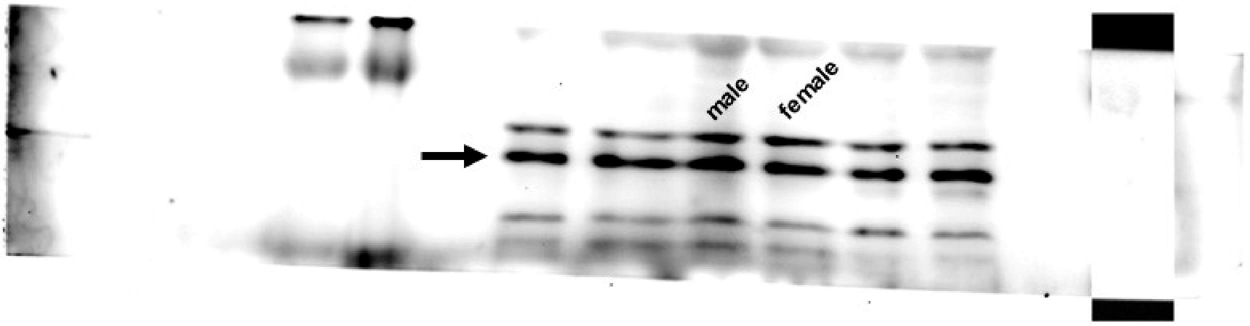

Supplementary Figure S2: Uncropped Western blots of VEGFR-1 and β-actin from whole cell extracts of islets.

Supplementary Figure S3

VEGFR-2

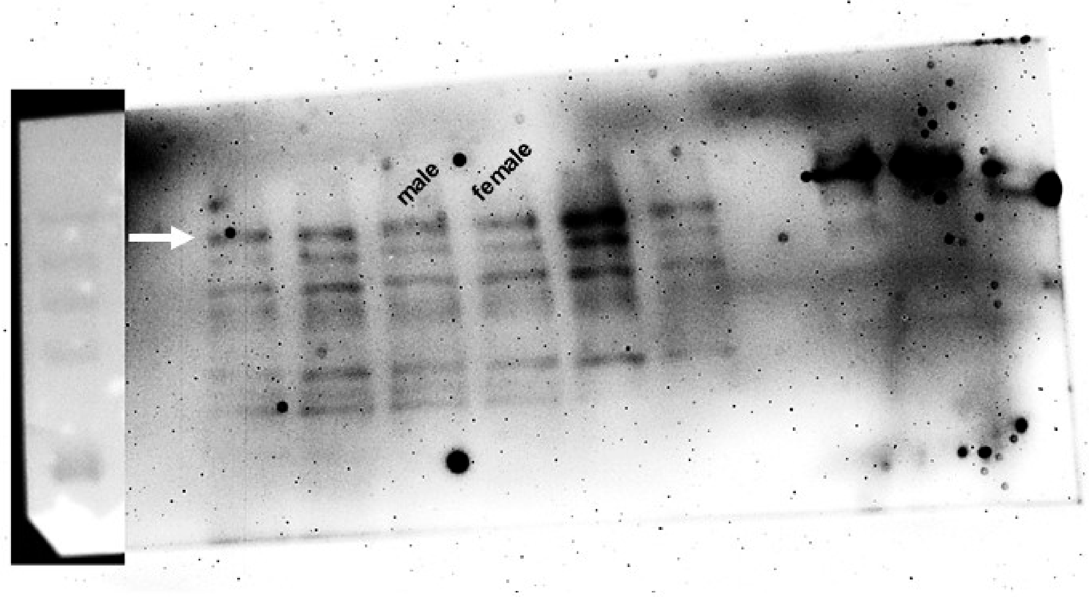

GAPDH

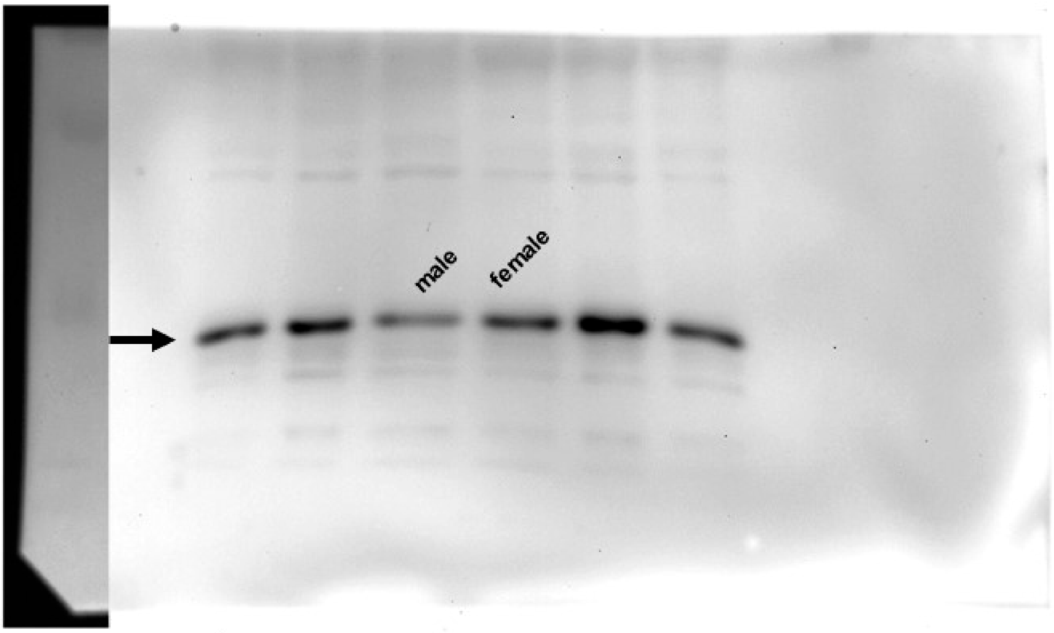

Supplementary Figure S3: Uncropped Western blots of VEGFR-2 and GAPDH from whole cell extracts of islets.

Supplementary Figure S4

CD31

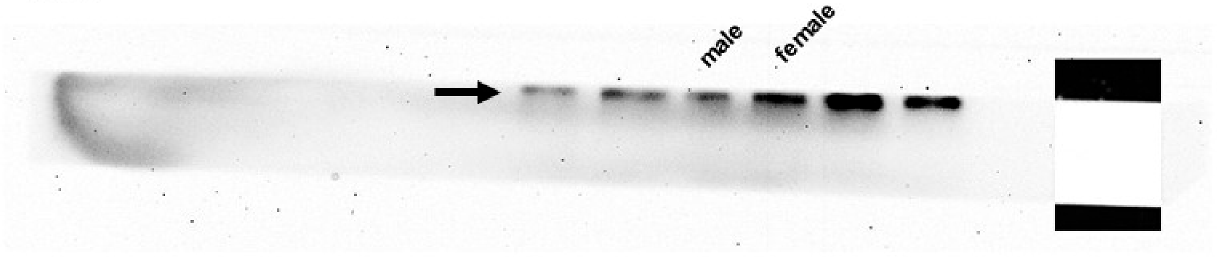

ß-actin

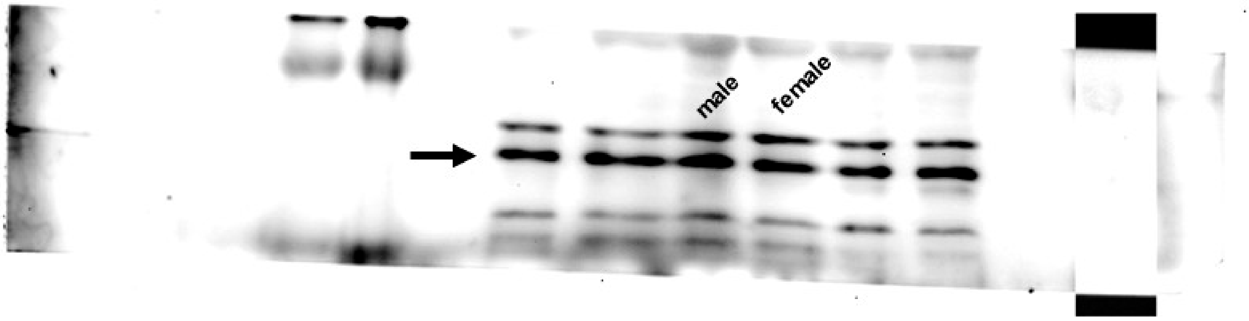

Supplementary Figure S4: Uncropped Western blots of CD31 and β-actin from whole cell extracts of islets.

Supplementary Figure S5

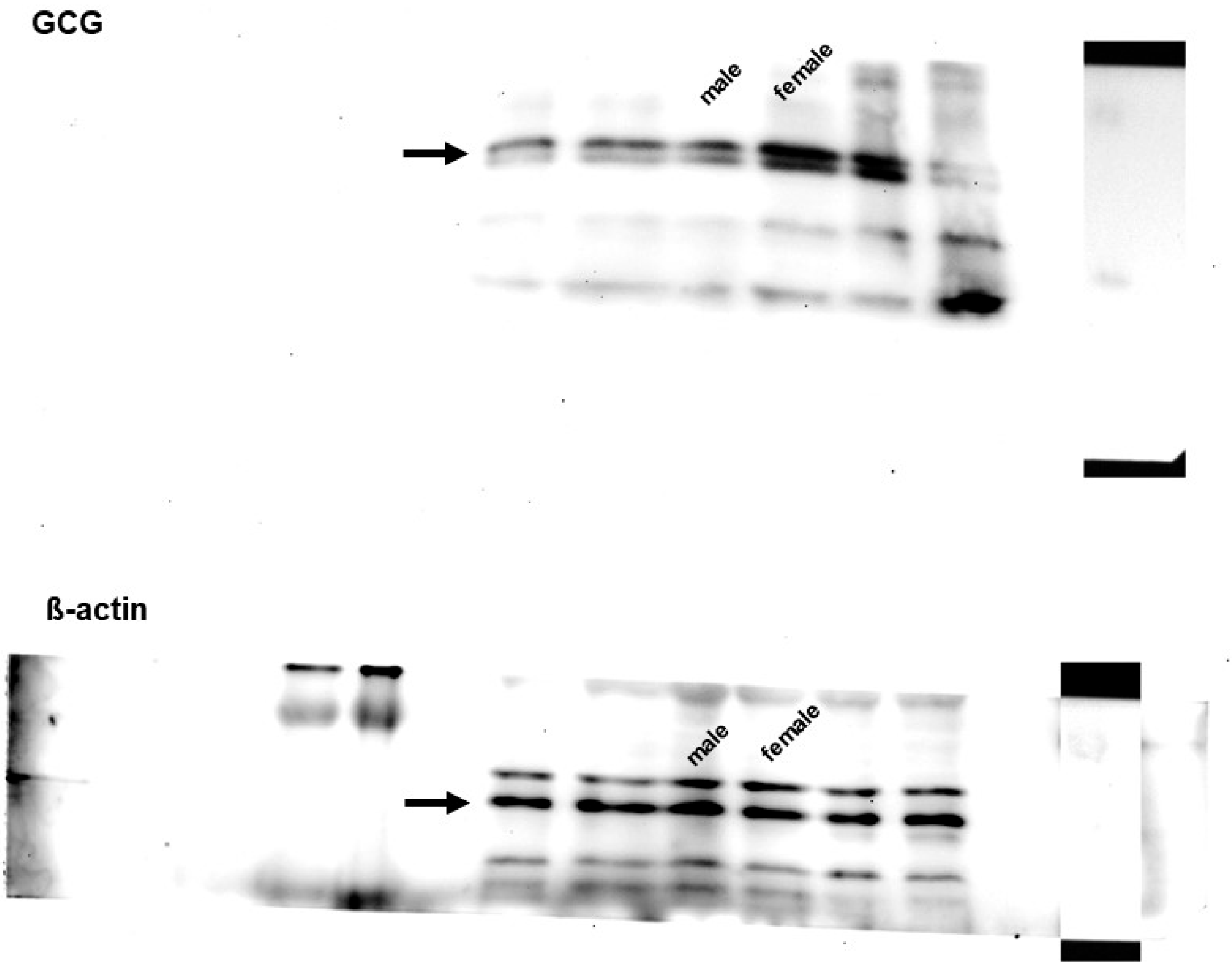

Supplementary Figure S5: Uncropped Western blots of GCG and β-actin from whole cell extracts of islets.

Supplementary Figure S6

GCGR

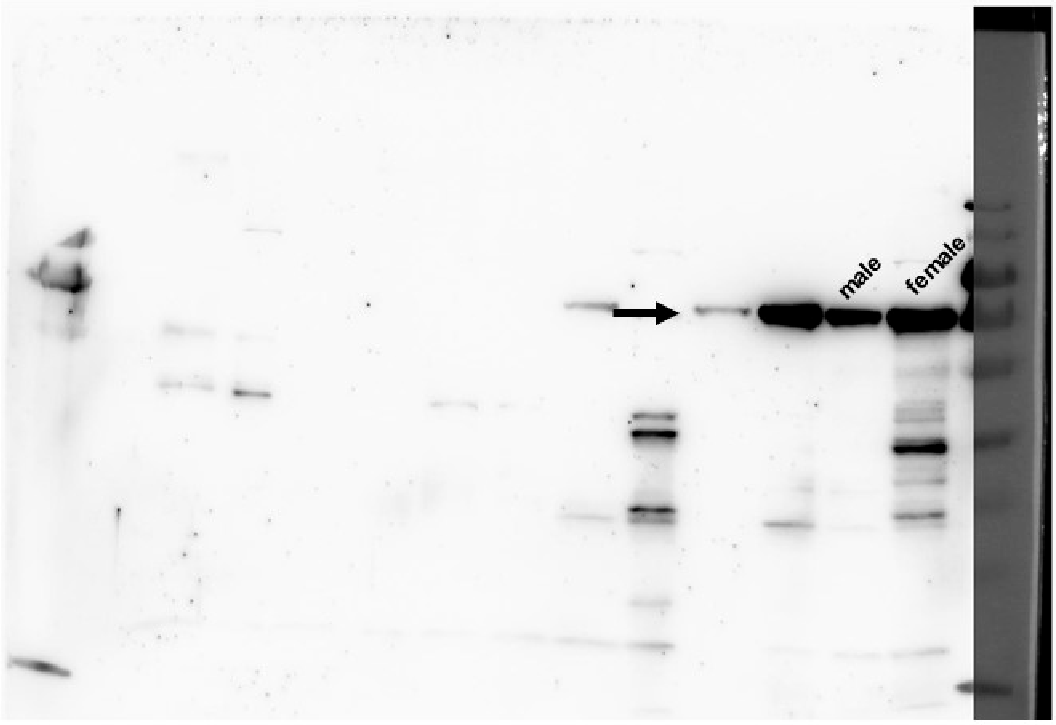

α-tubulin

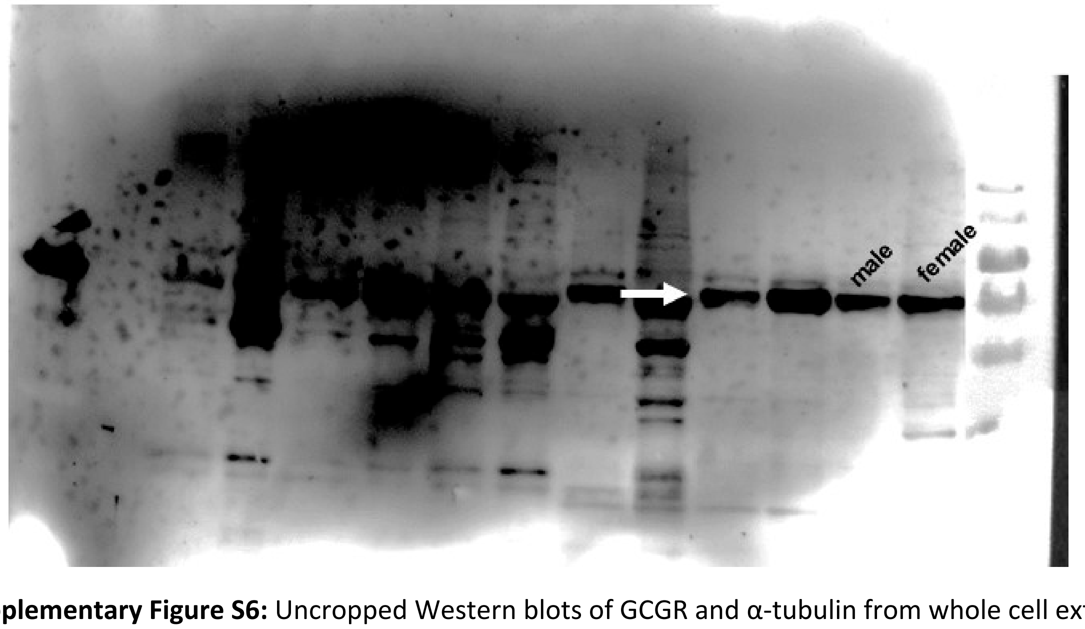

Supplementary Figure S6: Uncropped Western blots of GCGR and α-tubulin from whole cell extracts of islets.

Supplementary Figure S7

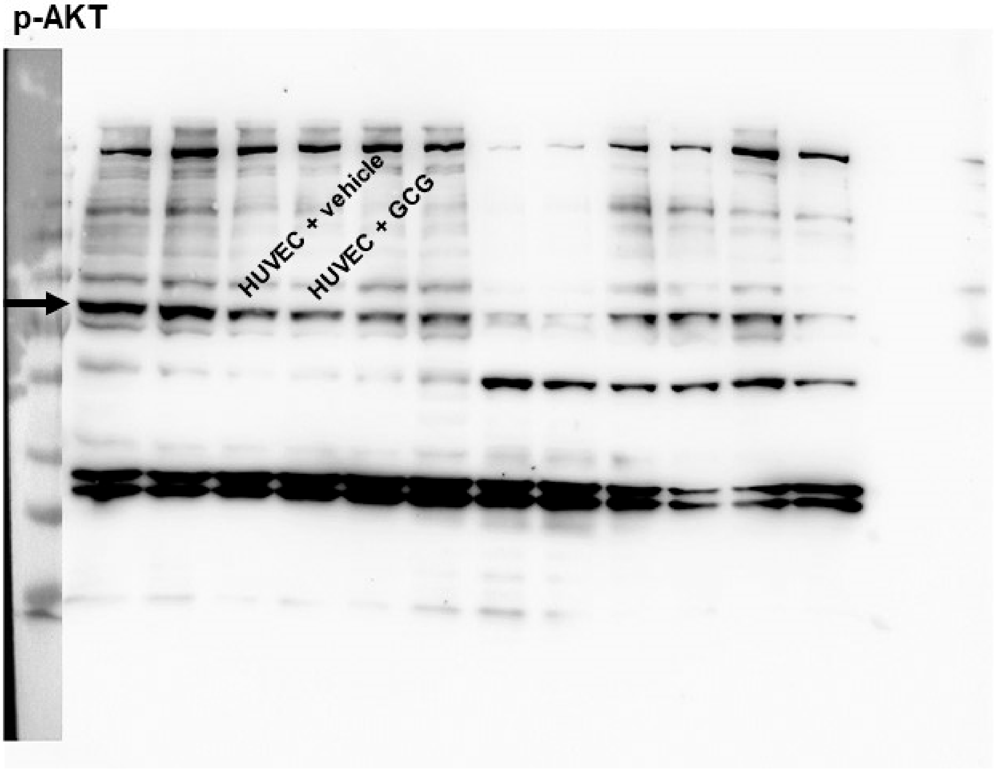

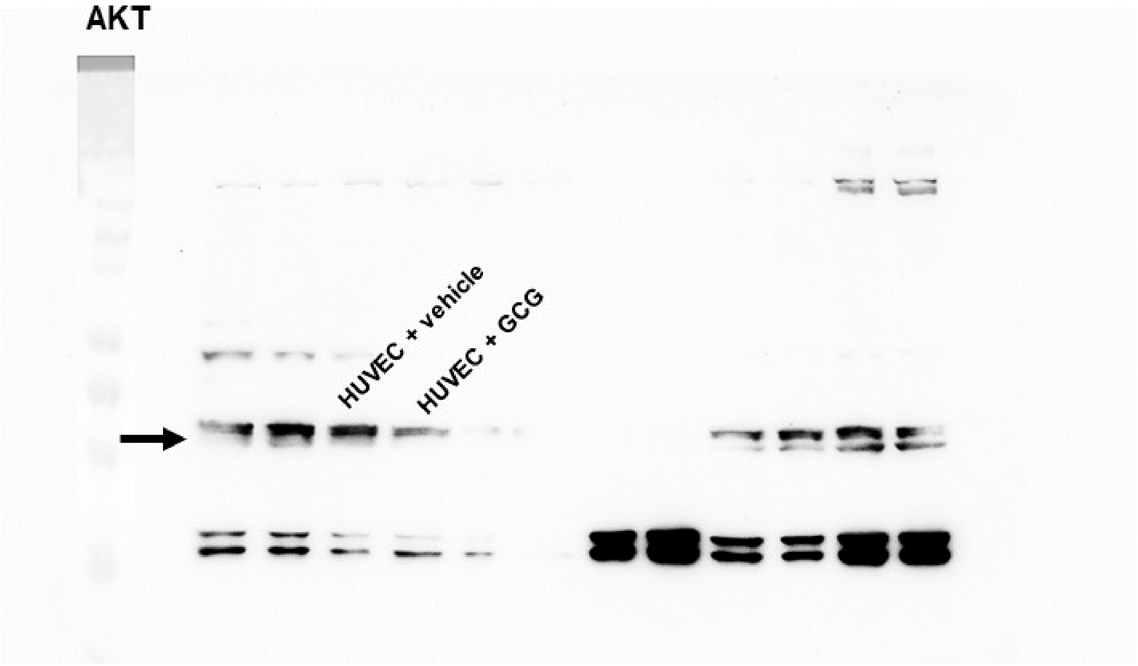

Supplementary Figure S7: Uncropped Western blots of p-AKT and AKT from whole cell extracts of HUVEC.

Supplementary Figure S8

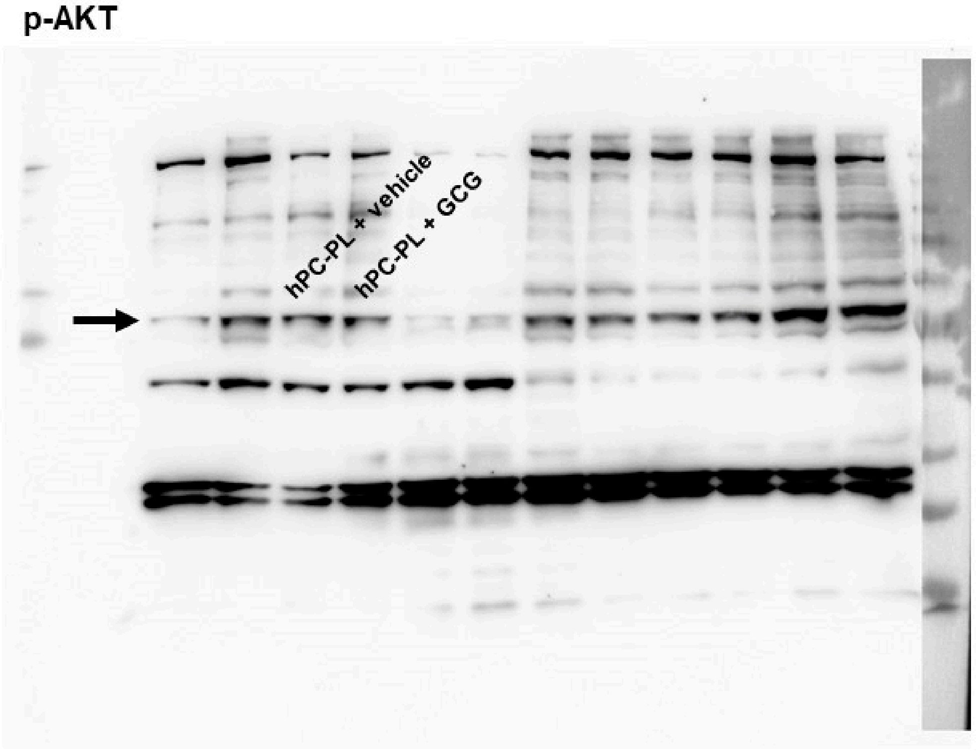

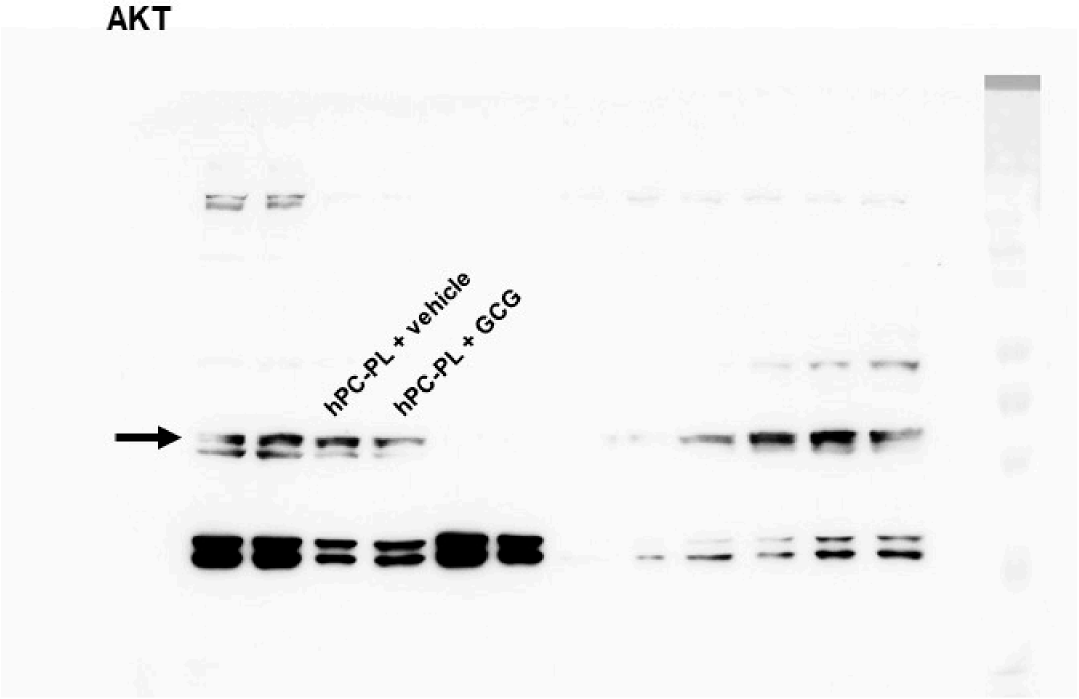

Supplementary Figure S8: Uncropped Western blots of p-AKT and AKT from whole cell extracts of hPC-PL.

Supplementary Figure S9

p-mTOR

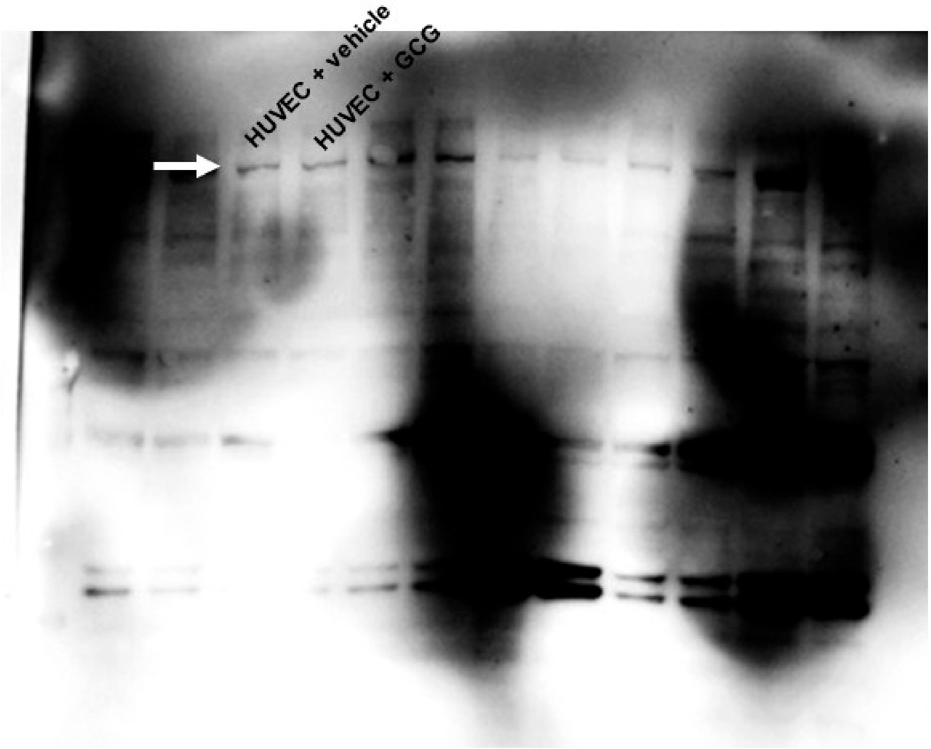

mTOR

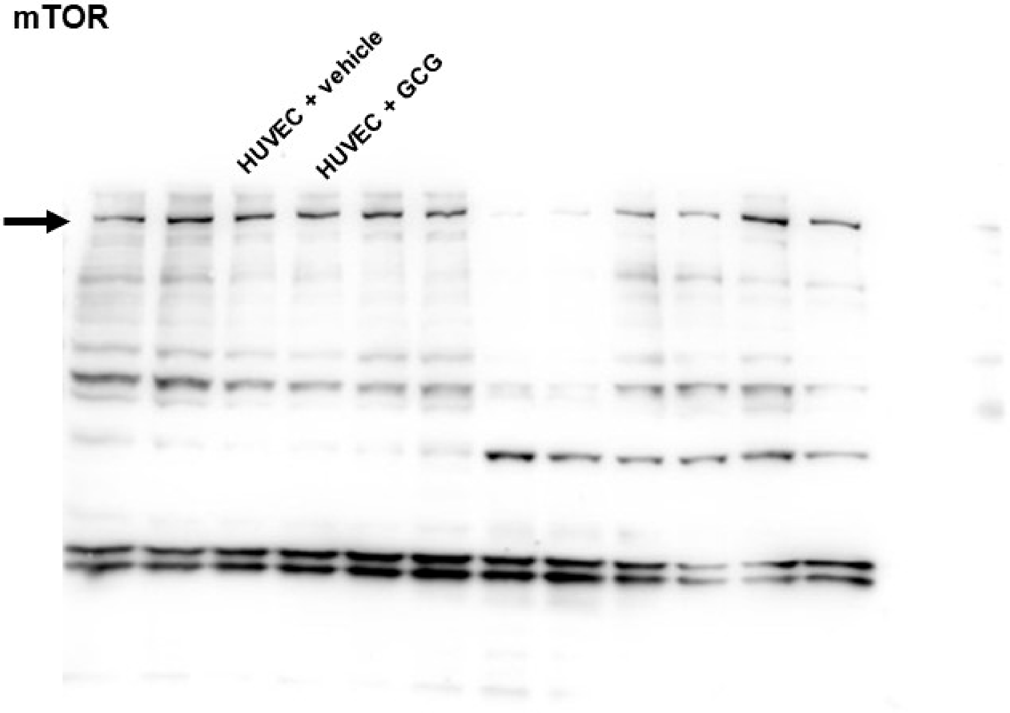

Supplementary Figure S9: Uncropped Western blots of p-mTOR and mTOR from whole cell extracts of HUVEC.

Supplementary Figure S10

p-mTOR

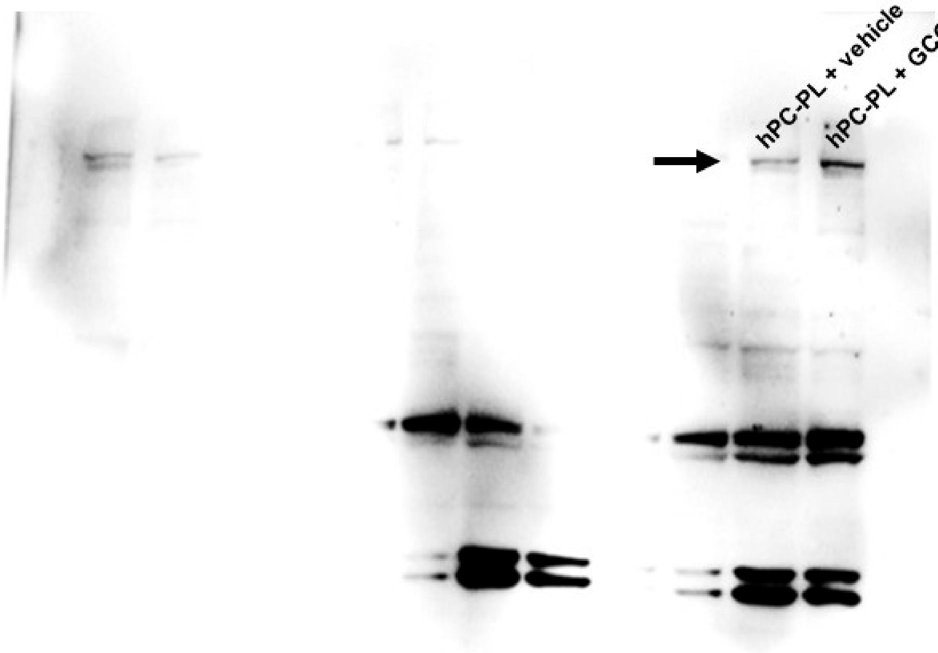

mTOR

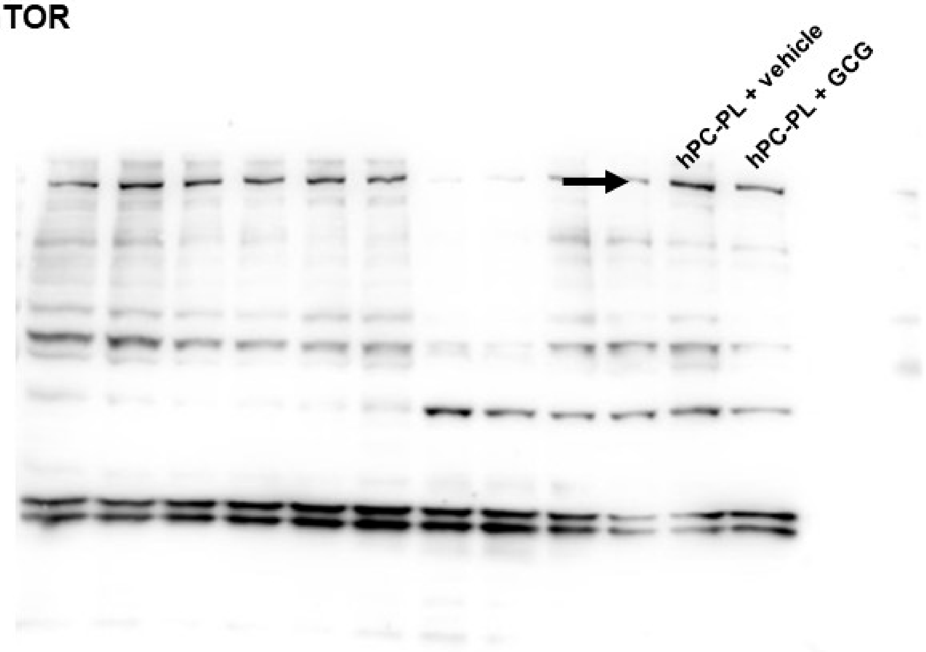

Supplementary Figure S10: Uncropped Western blots of p-mTOR and mTOR from whole cell extracts of hPC-PL.

Supplementary Figure S11

p-ERK

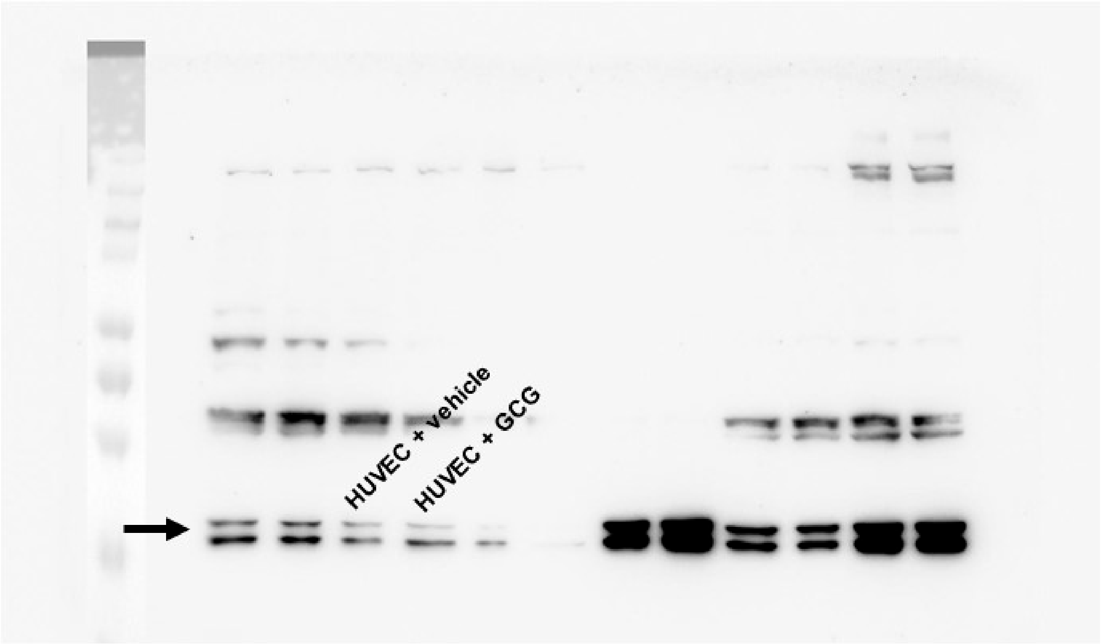

ERK

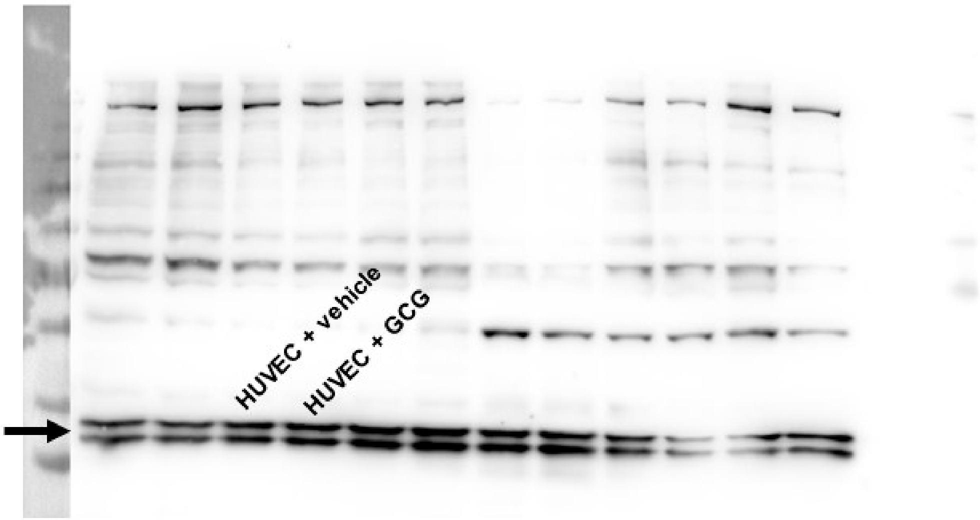

Supplementary Figure S11: Uncropped Western blots of p-ERK and ERK from whole cell extracts of HUVEC.

Supplementary Figure S12

p-ERK

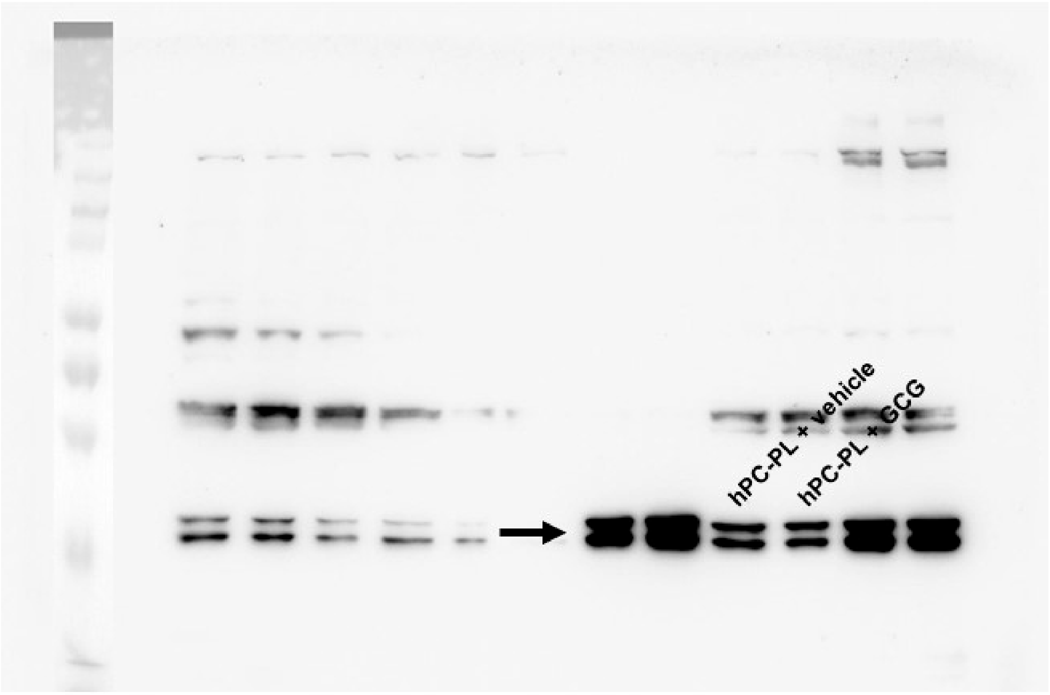

ERK

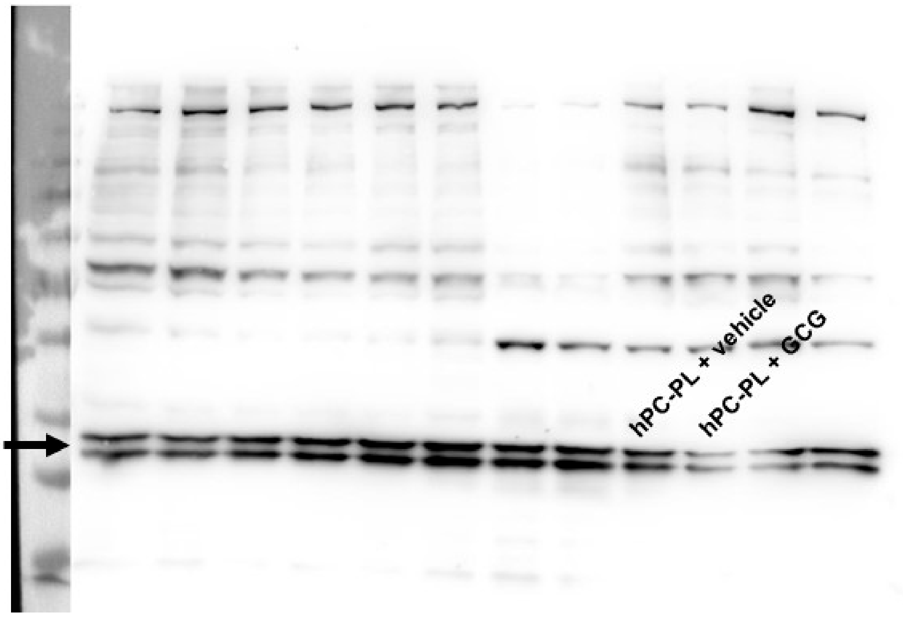

Supplementary Figure S12: Uncropped Western blots of p-ERK and ERK from whole cell extracts of hPC-PL.
